# Valence Change Bipolar Resistive Switching Accompanied With Magnetization Switching in CoFe_2_O_4_ Thin Film

**DOI:** 10.1038/s41598-017-12579-x

**Published:** 2017-09-29

**Authors:** Sandeep Munjal, Neeraj Khare

**Affiliations:** Department of Physics, Indian Institute of Technology Delhi, Hauz Khas, New Delhi, 110016 India

## Abstract

Resistive Switching in oxides has offered new opportunities for developing resistive random access memory (ReRAM) devices. Here we demonstrated bipolar Resistive Switching along with magnetization switching of cobalt ferrite (CFO) thin film using Al/CFO/FTO sandwich structure, which makes it a potential candidate for developing future multifunctional memory devices. The device shows good retention characteristic time (>10^4^ seconds) and endurance performance, a good resistance ratio of high resistance state (HRS) and low resistance state (LRS) ~10^3^. Nearly constant resistance values in LRS and HRS confirm the stability and non-volatile nature of the device. The device shows different conduction mechanisms in the HRS and LRS i.e. Schottky, Poole Frenkel and Ohmic. Magnetization of the device is also modulated by applied electric field which has been attributed to the oxygen vacancies formed/annihilated during the voltage sweep and indicates the presence of valence change mechanism (VCM) in our device. It is suggested that push/pull of oxygen ions from oxygen diffusion layer during voltage sweep is responsible for forming/rupture of oxygen vacancies conducting channels, leading to switching between LRS and HRS and for switching in magnetization in CFO thin film. Presence of VCM in our device was confirmed by X-ray Photoelectron Spectroscopy at Al/CFO interface.

## Introduction

The Resistive Random Access Memory (ReRAM) devices, based on the Resistive Switching (RS) phenomena, are emerging as a potential candidate for the next generation memory devices due to low power consumption^1^, high operation speed^2^, high-density integration^3^, non-destructive readout^4^, favourable scalability^5^ and good compatibility with complementary metal–oxide–semiconductor (CMOS) technology^6^. Besides memory applications, the ReRAM devices also offer a significant potential for use in the neuromorphic^7^ and logic applications^8^. The resistive RAM devices consist of a simple capacitor-like structure constituted of an insulating/dielectric layer between two metallic electrodes (MIM type structure)^9^ that exhibits reversible resistive switching on applying unipolar or bipolar voltages^10^. But, the mechanism behind the resistive switching phenomena still needs a great deal of discussion^11^. On the basis of the principle resistive switching mechanisms, ReRAM devices are classified into three categories (i) electrochemical metallization memory (ECM)^12^, (ii) thermochemical memory (TCM)^13^, and (iii) valence change memory (VCM)^14^. ECM is related to migration of metallic cations (e.g., Cu, Ag) in solid electrolyte thin film. TCM is related to change in stoichiometry due to increase of temperature induced by the electric current. On the other hand, VCM is triggered by oxygen anions migration and change in valence of the cations^15^. In VCM devices, the commonly observed conduction mechanisms are: (i) Ohmic conduction (ii) Schottky emission (iii) Poole Frenkel emission (iv) trap-assisted tunneling and (v) hopping conduction^16,17^. To enhance the performance of the memory device and for good data retention property, it is crucial to identify the exact transport mechanism and its relation with different resistive switching properties.

VCM is mainly found in oxide based ReRAM devices, in which oxygen vacancies play important role in the Resistive Switching phenomena, and generation/migration of these oxygen vacancies strongly depends upon the applied bias voltage and its polarity^18^. The oxygen vacancies are positively charged sites and can influence the physical properties of the material due to the redox reaction occurring in their vicinity^19^. It is well-known that in the magnetic oxides, the magnetic properties strongly depend upon the oxidation number of the cations and hence the magnetic behaviour of the material can also be tuned by controlling the migration of oxygen ions^20^. Using magnetic oxides in ReRAM devices can open a new door of memory devices where the magnetic properties of the device can also be modulated along with the resistive switching of the device. Although, electric field modulation of magnetization and RS effect in some magnetic oxides has been investigated, but the mechanism of RS effect and its correlation with the magnetization have not been fully understood^11^. Xiong *et al*.^21^ investigated the RS effect and change in the magnetization in the La_2/3_Ba_1/3_MnO_3_ and attributed the switching effect to the break or repair of the -Mn^3+^-O^2−^-Mn^4+^- chains induced by the electric field through the oxygen vacancies migration. Ren *et al*.^22^ attributed the RS effects and associated change in magnetization in Ag/Ti/Fe_2_O_3_/Pt device to the valence change in Fe ion, whereas Chen *et al*.^23^ attributed to RS effects in Fe_2_O_3_/Nb:SrTiO_3_ heterojunctions to the carrier injection. In Pt/NiFe_2_O_4_/Pt device, formation of conductive filament have been attributed to the occurrence of the unipolar RS effect^24^. Bipolar Resistive Switching memory devices are considered to be better than Unipolar Resistive Switching devices in terms of data retention, device consistency, controllability and storage performance^25^. Most of the reports on RS properties of stoichiometric magnetic oxides have used noble (chemically inert) material as an electrode, which generally leads to a unipolar behaviour of the device^24,26–28^. However, using a chemically active material as an electrode can lead to bipolar type of RS and can enhance the performance of the RS device.

CoFe_2_O_4_ (CFO), a ferrite with inverse spinel structure^29,30^, is another very interesting magnetic oxide, which has attracted lots of interest due to its potential applications such as magnetoelectric coupling^31^, spin filtering^32^, microwave absorber^33–35^ and magnetic hyperthermia^36^. There have not been any detailed investigation of assessing the potentiality of CFO magnetic oxide for the multifunctional memory devices. Using cobalt ferrite as RS active material and understanding the exact mechanism can provide an extra degree of freedom to manipulate the magnetic properties of these materials using electric field. Herein, we present a multifunctional Resistive Switching memory device using CFO thin film with aluminum (a chemically active metal) as top electrode and having a simple structure (Al/CoFe_2_O_4_/FTO(fluorine doped tin oxide)), whose magnetic properties can also be modulated along with the switching in the resistance states only by voltage stimulus. The SET and RESET processes occur in a more uniform and stable manner with a narrow switching voltage distribution at comparatively smaller electric fields compared to previous reports^26,27^. The underlying switching mechanism is discussed on the basis of electric/magnetic properties of the device in different states (high resistance state and low resistance state). A possible model for explaining the behavior of the device in different states have also been proposed.

## Results and Discussion

**Figure 1:**
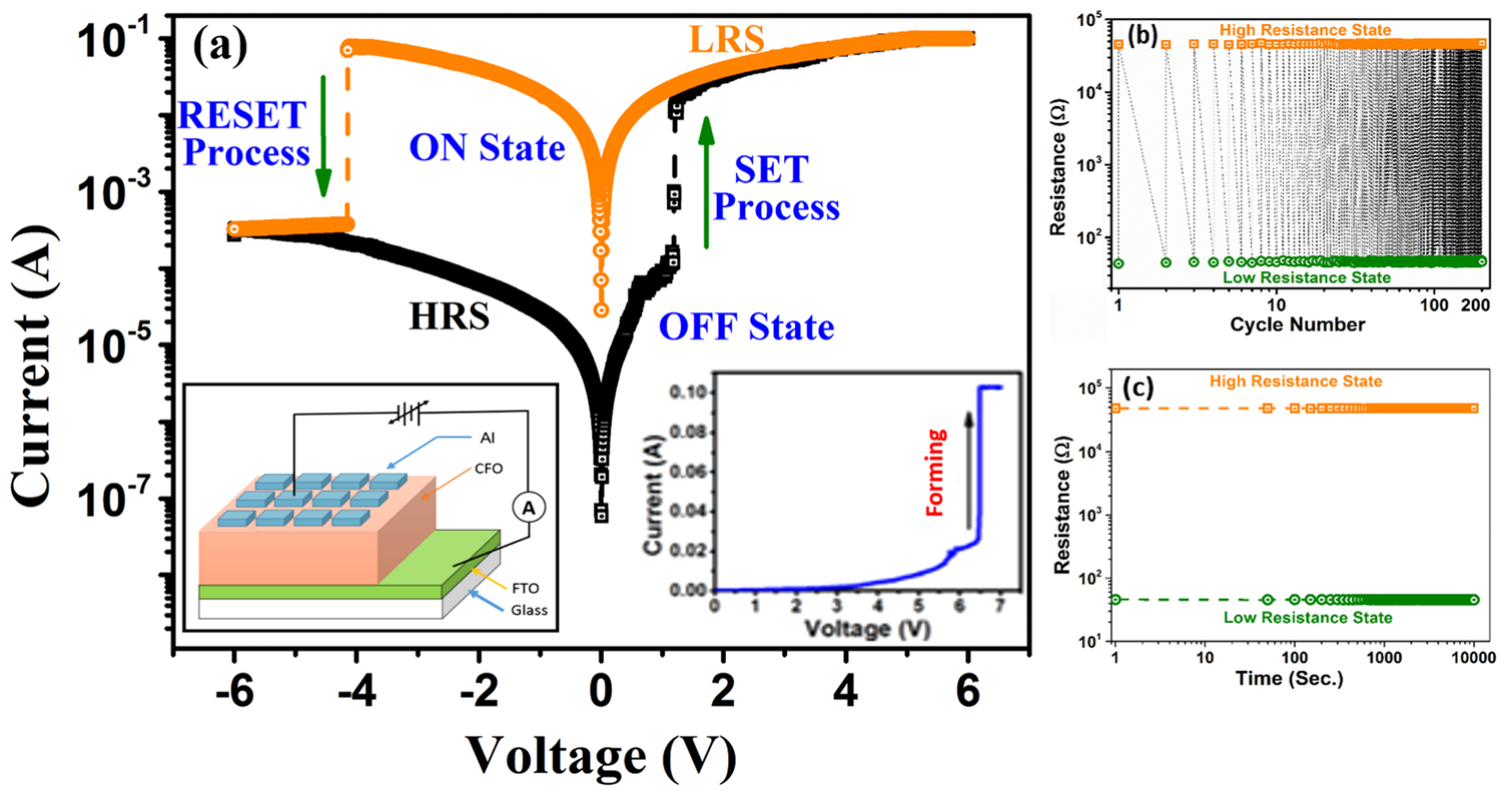
(**a**) Current–voltage (I–V) curve of resistive memory device in Al/CoFe_2_O_4_/FTO structure. The device is electroformed by applying a relatively high positive bias on Al electrode and resulting I–V curve is shown in the right inset. The left inset shows the schematic of the fabricated ReRAM Al/CoFe_2_O_4_/FTO Resistive memory cell device. (**b**) The endurance performance and (**c**) Retention property of Al/CoFe_2_O_4_/FTO structure read at 0.1 volt, at Room temperature.

Resistive Switching properties of cobalt ferrite (CFO) thin film were studied by fabricating Al/CoFe_2_O_4_/FTO type M/I/M structure. The schematic of the Al/CFO/FTO sandwich type device is shown in the left inset of Fig. 1(a). The current-voltage (I–V) characteristic curve of the Al/CFO/FTO device is shown in Fig. 1(a). It is clear from the I–V curve that the device shows reversible, nonvolatile bipolar resistive switching. The fabricated device shows very high resistance in its pristine state (PS) and electroforming process was found necessary. The right inset of Fig. 1(a) shows the I–V curve of the electroforming process. During the electroforming process (at ~6.5 volt) the device switches from pristine state to low resistance state (LRS). After the electroforming process the voltage was swept between +5 and −5 volts in a cyclic manner. In Fig. 1(a), the arrows indicate the direction of voltage sweep. During this voltage sweep, the resistance of the device increases suddenly at the voltage near −4 volt and this transition takes the device to High Resistance State (HRS). In the positive voltage sweep, the resistance of the device suddenly decreases near 1.1 volt and the device switches into Low Resistance State (LRS) from HRS. The LRS state is also known as “ON” state or “1” state, similarly the HRS state is termed as “OFF” state or “0” state^37^. The ratio of resistance of HRS and LRS of the device has been observed as ~10^3^, which indicates that the present device shows good switching in resistance in positive and negative voltage directions. To perform the endurance tests, the I–V measurements of the device in the cyclic voltage sweep are repeated 200 times and the resistance in HRS and LRS at read voltage of 0.1 volt with cycle number is shown in Fig. 1(b). The endurance performance of the fabricated device demonstrates a very good Bipolar Resistive Switching over 200 cycles. Further, to test the stability of resistance states of the device with the time a retention test was also performed. The result of retention capability test at readout voltage 0.1 volt is shown in Fig. 1(c). Constant values of resistance in LRS and HRS state over a long period of time ~10^4^ s confirm the stability and non-volatile nature of the device.

**Figure 2:**
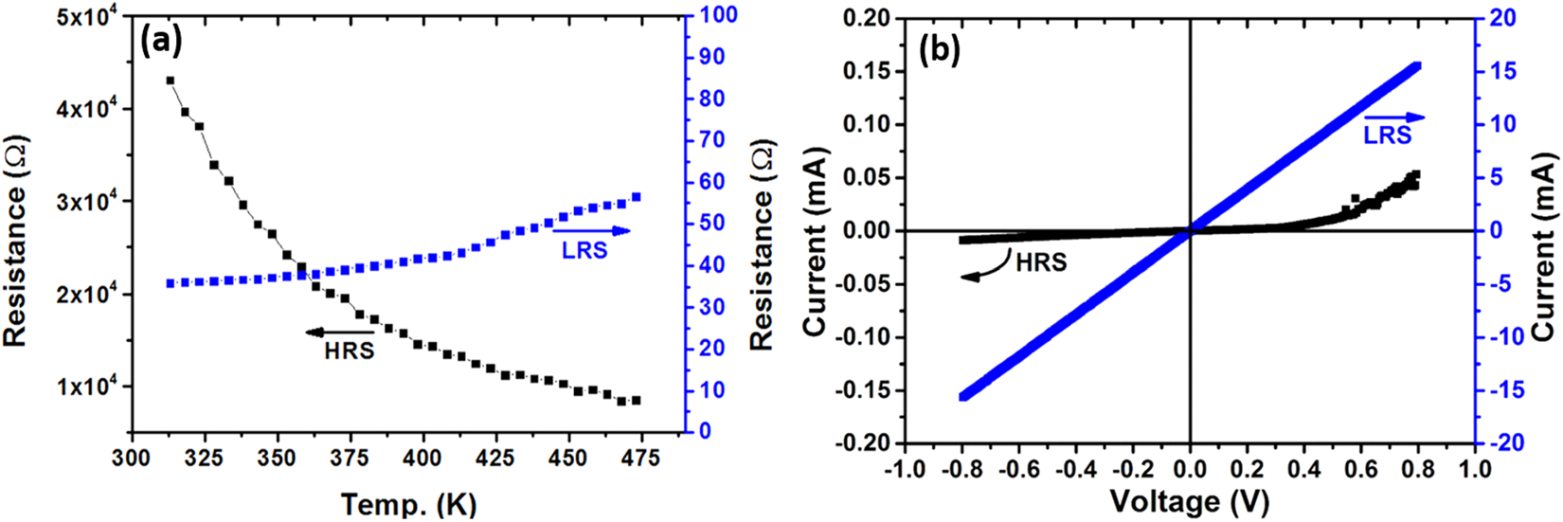
(**a**) The Resistance vs. Temperature curves for Low Resistance State (blue) and High Resistance State (Black). (**b**) Voltage vs. Current characteristic curves in low voltage range for Low Resistance State (blue) and High Resistance State (Black).

**Figure 3:**
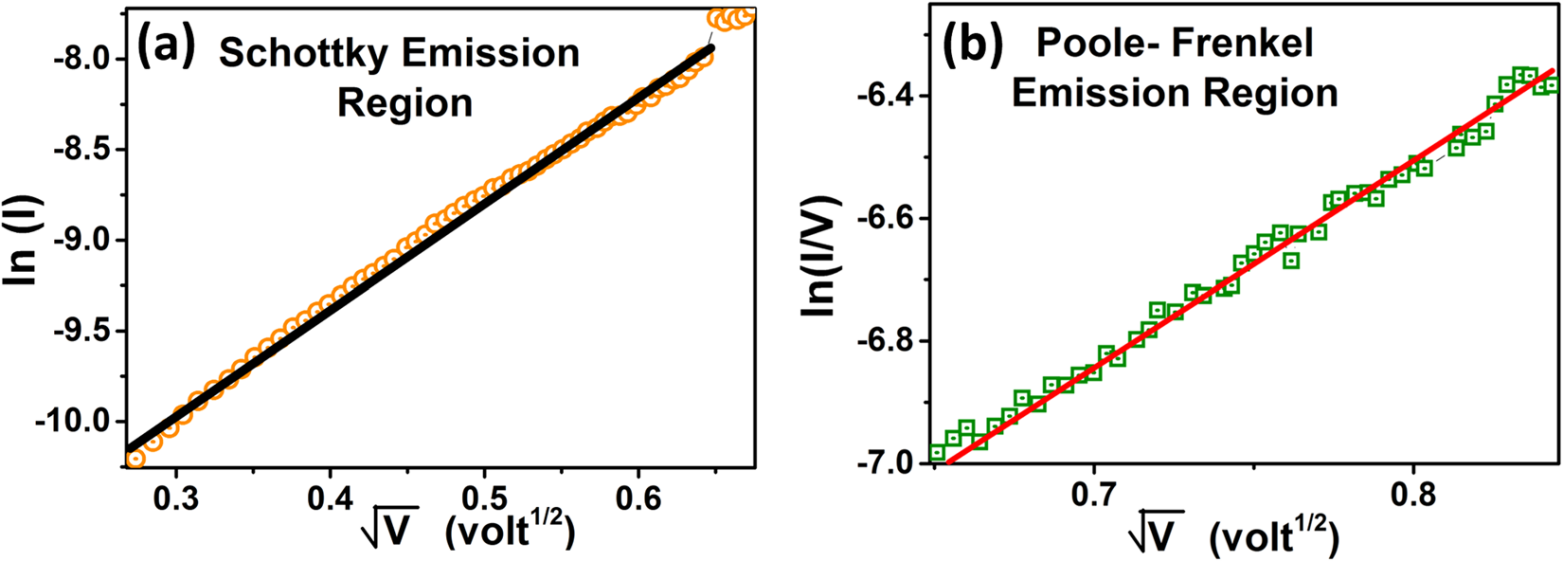
Current-voltage characteristic curves showing (**a**) Schottky emission and (**b**) Poole Frenkel emission in High Resistance State in different applied bias voltage regions.

For understanding the switching mechanisms of Al/CFO/FTO device, temperature dependence of the resistance (R-T) of the device and I–V characteristics were studied when the device was in low resistance state and at high resistance state. Figure 2(a) shows the results of the R-T studies at 0.1 applied bias. In the low resistance state the device exhibit metal like behavior whereas in the high resistance state a semiconducting behavior was observed. By fitting the temperature dependence of resistance in HRS to the Arrhenius equation R = R_0_.e^−∆E**/**kT^, the thermal activation energy was estimated as ~129 meV. In order to explore further the conduction mechanism of the device in LRS and HRS, I–V characteristics of the device were investigated (Fig. 2b). A linear I–V characteristics of the device in the LRS confirms the ohmic behavior, whereas asymmetric nonlinear I–V characteristics in HRS indicate the presence of a barrier on the Al/CFO interface. The nonlinear I–V characteristics in HRS of the device indicates that the conduction mechanism can be Schottky emission^38^, Poole Frenkel conduction^39^, or space charge limited current (SCLC) conduction^40^. These different conduction mechanisms can be identified from their specific voltage dependence on the current. For schottky emission, ln(I) ∝ V^0.5^, whereas for the Poole Frenkel conduction and space charge limited current conduction, the current voltage characteristics are expressed as ln(I/V) ∝ V^0.5^ and I ∝ V^2^ respectively^41,42^. For the present Al/CFO/FTO RS device in the HRS, the current did not follow the square dependence for the applied voltage, which ruled out the presence of space charge limited current conduction. For investigating the presence of Schottky emission or Poole Frenkel conduction, we plotted the I–V curves as ln(I) vs. V^0.5^ and ln(I/V) vs. V^0.5^ curves (Fig. 3). It is evident from the Fig. 3(a) that the ln(I) vs. V^0.5^ curve can be fitted with a straight line only for the variation of the voltage from 0.08 V to 0.41 V, whereas the ln(I/V) vs. V^0.5^ curve (Fig. 3(b)) can be fitted with a straight line from 0.41 V to 0.71 V. This suggests that the conduction mechanism in the high resistance state of the CoFe_2_O_4_ device is initially dominated by the Schottky emission and afterward at higher bias voltage (V > 0.41 volt), it is dominated by the Poole Frenkel conduction. The presence of such type of two conduction mechanisms dominating at different bias voltages has been reported earlier also in different resistive switching devices^43–48^.

In pristine state, as the positive voltage on the top Al electrode is increased, at a sufficiently high applied voltage, the negatively charged oxygen ions start migrating from the highly insulating CFO active layer toward the top electrode, resulting in the formation of conducting channels of oxygen vacancies, connecting the bottom electrode and top electrode. This transition takes the device into low resistance state and the maximum current in this process is limited by the compliance current. Whereas, in the reset process (negative bias) the electric field pushes the oxygen ions toward the active layer of CFO and, the concentration of oxygen vacancies decreases enough, that may rupture the conduction path and the device transforms into HRS. In HRS, the conduction mechanism below 0.41 volt is dominated by the Schottky emission, whereas, in high electric field range the conduction is dominated by Poole Frenkel emission which may be attributed to the electric field-assisted thermal excitation of trapped electrons into the conduction band and the trapping centers provided by the defects after a sufficiently higher applied electric field. On increasing the applied voltage further, to set voltage the oxygen vacancies are formed again and forced to align to reconstruct the conducting channels, leading to the transition from Poole Frenkel emission to Ohmic conduction.

**Figure 4:**
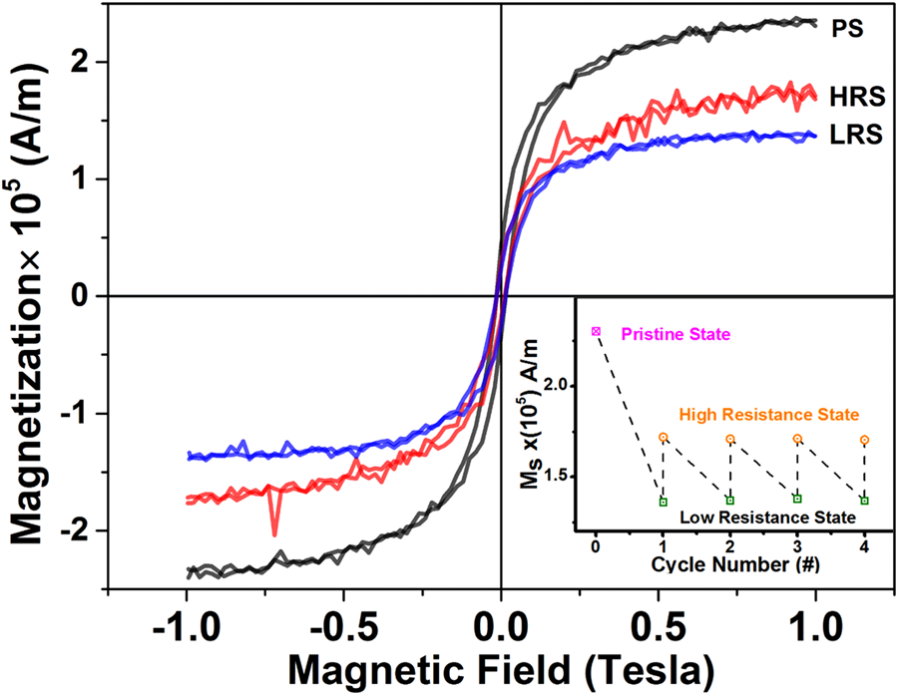
Magnetic hysteresis loops of the fabricated Al/CoFe_2_O_4_/FTO device in different resistance states i.e. Pristine State, Low Resistance State and High Resistance State. The inset of the image shows the variation of saturation magnetization in pristine state, Low resistance state and High resistance state with cycle number.

To understand the exact role of oxygen vacancies in the bipolar resistive switching mechanism in our device, we investigated the magnetic properties of the device in the Pristine State (PS), Low Resistance State (LRS) and High Resistance State (HRS). Figure 4 shows magnetization vs. magnetic field (M-H) loops for the device in different resistance states at 300 K. The device is observed to exhibit ferromagnetic behavior. The saturation magnetization (Ms) of the sample in pristine state (PS) was greater than that of the HRS sample, and Ms of the sample in HRS was greater than that of the sample in LRS. We repeated the magnetization measurements with the resistive switching cycle. The inset of the Fig. 4 shows the variation of saturation magnetization in pristine state, Low resistance state and High resistance state with cycle number. Periodic modulation of M_s_ of the material with the applied electric field and synchronization of the variation of the M_s_ with LRS and HRS clearly indicates that for the present device of CFO, the magnetic properties can also be switched along with the change of the resistance state. As we are observing a change in magnetization also along with the resistive switching which suggests that conducting channels made of oxygen vacancies formed between top and bottom electrodes cannot be highly localized in nature and a number of oxygen vacancies must be created on applying the positive bias on the top electrode during the electroforming process providing the direct conducting path between the electrodes. The voltage stimulus control over the magnetic properties can offer additional degrees of freedom for CFO based multifunctional devices.

The modulation of the magnetic properties of the Al/CFO/FTO device with the resistance state may be attributed to the difference in the concentrations of oxygen vacancies. These oxygen vacancies may reduce some of the Co^2+^ ions, leading to the formation of Co^0^; or, it is also possible that the oxygen vacancies may reduce some of the Fe^3+^ ions to Fe^2+^ ions, changing the valence of some iron ions. In the CoFe_2_O_4_ inverse spinel structure, half of the Fe^3+^ ions occupy “A” sites and the other half of the Fe^3+^ ions are in “B” sites, whereas the Co^2+^ ions resides in the “B” sites^49^. The net magnetization of CFO is given as; M_S_ = M_B_-M_A_, where M_A_ and M_B_ are net magnetic moments at “A” and “B” sites^50^. The relative change in magnetic moments of site “A” and “B” can alter the net magnetic moment of cobalt ferrite^51^. There can be two possible cases: the oxygen vacancies can reduce the cations present in (i) “A” site or (ii) “B” site. The first possibility of reduction of cations at “A” site, by oxygen vacancies can be ruled out, as this should increase the net magnetic moment of the sample, which contradicts our results. However, in the latter case, the total magnetic moment is expected to decrease, which is in agreement with our magnetization results. Therefore, the oxygen vacancies can reduce Co^2+^ ions to Co^0^ and Fe^3+^ ions to Fe^2+^ ions, that is, the valences of the cations present in “B” sites is changed by the influence of these oxygen vacancies.

The interatomic distance of Fe–O is shorter than Co–O due to smaller atomic radius of Fe^3+^ compared to that of Co^2+^ ^52^. Theoretical calculations also showed that Fe-O bonds are stronger than Co-O bonds, that is, the formation energy of oxygen vacancy near Fe^3+^ ions is larger than that near Co^2+^ ions, suggesting that oxygen vacancies near Co^2+^ ions would form more easily in CFO films at B site. The formation of oxygen vacancies may reduce Co^2+^ ions to Co atom losing magnetism, and thus the saturation magnetization of the CFO film would decrease. Conversely, when the CFO heterostructure is in HRS and reverse bias voltage is applied, the oxygen ions would move toward the opposite direction and refill in the original lattice position, which makes oxygen vacancies decrease, Co atoms are oxidized to Co^2+^, and the saturation magnetization of the CFO film increases. This magnetic modulation becomes a strong evidence of existence of valence change mechanism of resistive switching in our fabricated device.

**Figure 5:**
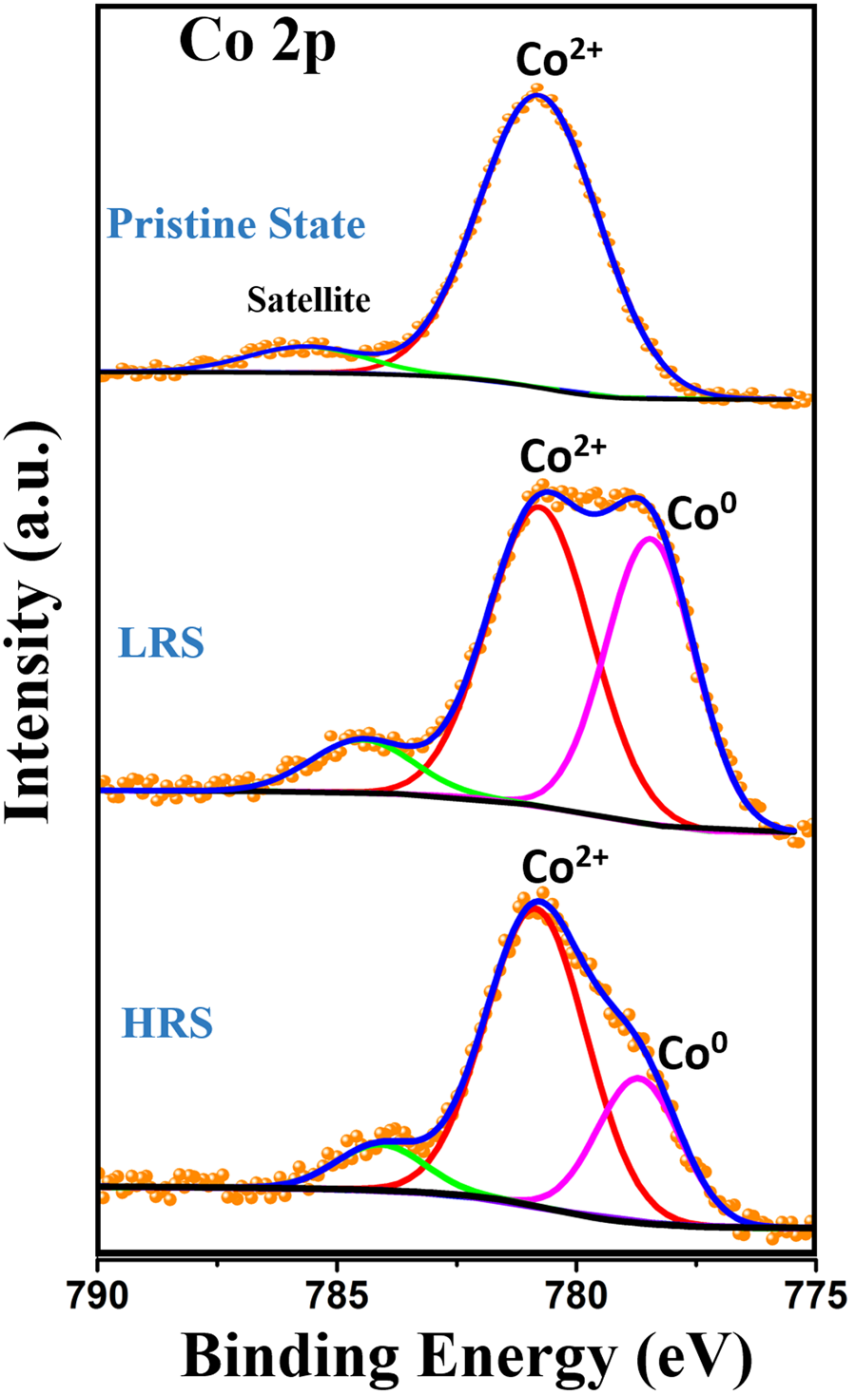
XPS spectra of Co 2p core level at the Al/CFO interface of the device.

It is known that X-ray photoelectron spectroscopy (XPS) is sensitive to chemical environment of atoms, which can provide information for the change of chemical state during the RS effect^22,28,53,54^. We have conducted XPS studies of Al/CFO/FTO device in Pristine State as well as in Low Resistance State and High Resistance State after electroforming and the results show the valence change of cobalt during the resistive switching. XPS spectra was recorded near the Al/CFO interface in the range of 0–1100 eV after sputter-etching the top electrode (etching rate ∼4 nm min^−1^) with Ar^+^ ions (2 kV, emission current 2 µA). The binding energy scale of the spectra was calibrated for the charging effect with respect to C 1 s peak (284.6 eV) corresponding to adventitious carbon present on the sample surface. The Co 2p spectra taken from the Al/CFO interface of Al/CoFe_2_O_4_/FTO device after sputtering etching the top Al electrode in Pristine, Low Resistance and High Resistance States of the device are shown in Fig. 5. The Co 2p XPS scans for the device in pristine state are fitted by two Gaussian peaks arising from Co^2+^ at 780.8 eV and one satellite peak around 785.8 eV^55^. A shift of the Co 2p XPS spectrum toward low binding energy was observed in the switched devices. The Co 2p peaks of the LRS and HRS device can be fitted by Co^2+^ (780.8 eV) and Co^0^ (778.6 eV) and one satellite peak^55^. This indicates a partial reduction of Co^2+^ ions to Co^0^ and confirms the presence of valence change mechanism behind the resistive switching phenomena present in our fabricated device. The ratio of the area of Co^2+^ and Co^0^ peaks changes when the device switches between LRS and HRS which confirms valence change of cobalt during the resistive switching process. No significant change was observed in the Fe 2p XPS spectrum of the device in pristine, Low Resistance and High resistance state (see supplementary information). This clearly indicates that no change in the valence of Fe has taken place during the resistive switching. This may be attributed to shorter bond length of Fe–O bond compared to Co–O due to smaller atomic radius of Fe^3+^ than Co^2+^, which makes the Fe-O bond stronger than Co-O and the easy formation of oxygen vacancies near Co^2+^ ions which further reduces the Co^2+^ ions to Co^0^ atom losing the magnetic characteristics^52^.

We have performed experiments to see the dependence of resistance of the device in LRS and HRS on the area of the top Al contact pad. We observed that the resistance of the device in LRS and HRS is independent of the contact pad area (see supplementary information). This confirms that the resistive switching in our fabricated Al/CoFe_2_O_4_/FTO is not of interface type. Further, the resistance of Al/CFO/FTO device and magnetization changes significantly with the resistive switching indicating the formation of a large number of oxygen vacancies in electroforming process and push/pull of these oxygen vacancies during the resistive switching process which suggests the formation of a broader sized conducting channel formed of oxygen vacancies instead of a highly localized filament. It is to point out that the similar results have been observed by Shen *et al*. for W/BST/SRO (W/BaSrTiO_3_/SrRuO_3_) device, where the improved resistive switching was attributed to WO_x_ interfacial layer but a filamentary type conduction was proposed as the resistance of the device was independent of contact pad size and it is suggested that the location of the switching is not exactly at the top interface^56^.

**Figure 6:**
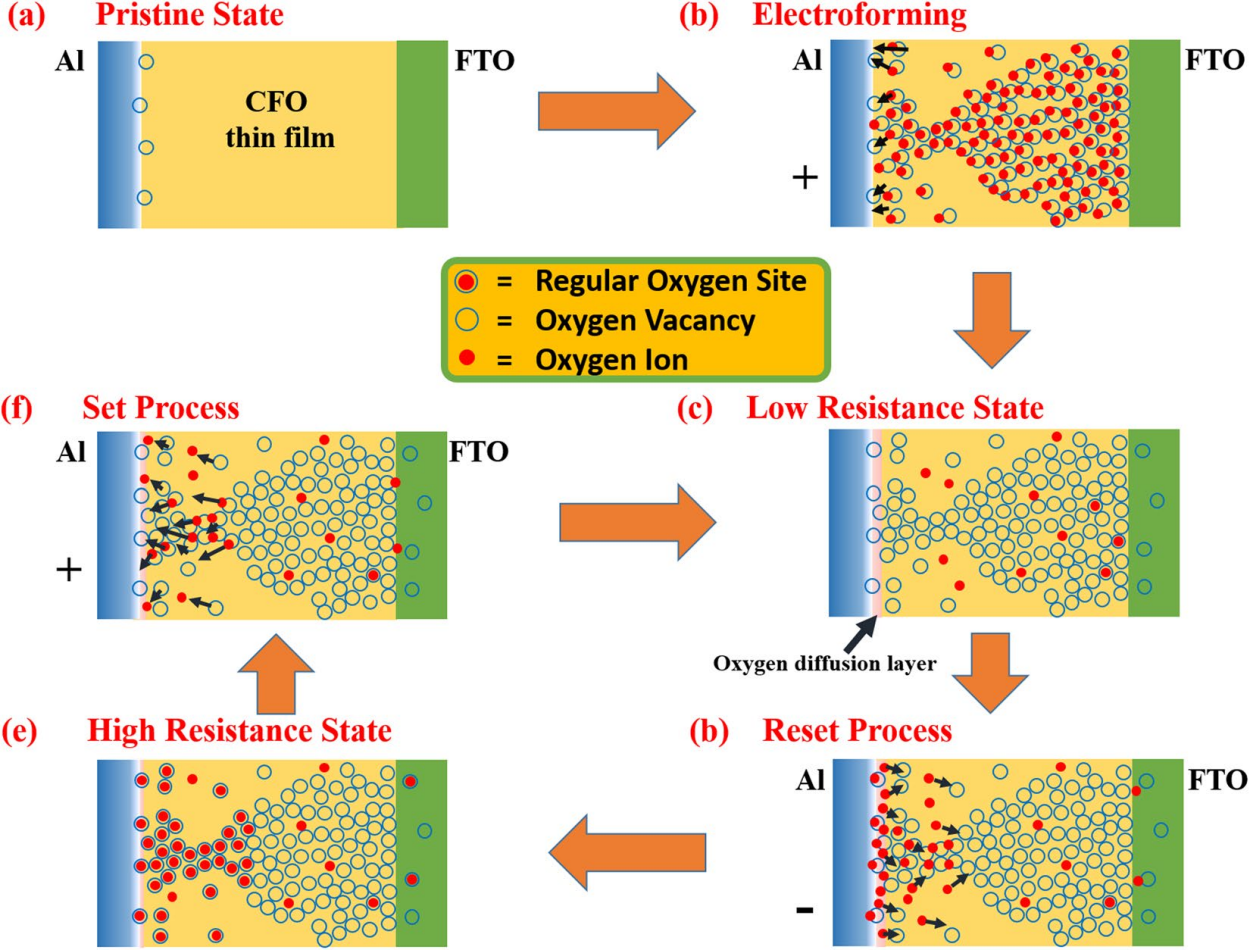
Schematic representation of (**a**) Pristine state with top (Al) and bottom (FTO) electrodes, (**b**) Electroforming process, (**c**) Low resistance state with a conducting channel in CFO layer and oxygen diffusion layer near Al electrode, (**d**) Reset process, when oxygen ions are coming back from oxygen diffusion layer to CFO layer, (**e**) and (**f**) High resistance state and Set process.

Figure 6 shows the schematic representation of the proposed model for driving mechanism to explain the occurrence of resistive switching as well as change in the magnetization state of the Al/CFO/FTO device. Figure 6(a) is the pristine state of the device that shows the CoFe_2_O_4_ layer is sandwiched between the two electrodes FTO and Al respectively. In this state the CoFe_2_O_4_ layer is highly resistive and no significant current flows through it until a higher electric field is applied to it for electroforming. As the top Al electrode is an active electrode, some oxygen vacancies can be generated near the top Al electrode as fabricated which was also confirmed by X-ray photoelectron spectroscopy (XPS) measurements (see supplementary information). On applying positive bias, at the anode, an electrochemical reaction occurs that separates the oxygen ions from the regular oxygen sites and these oxygen anions can move under the effect of applied electric field which is equivalent to the drift of oxygen vacancies^57^. This electrochemical reaction can be written in Krὅger-vink notations^58^ as;

1 $${{\bf{O}}}_{{\bf{o}}}\to {{\bf{V}}}_{{\bf{o}}}^{++}+{{\bf{O}}}^{-{\bf{2}}}({\rm{Toward}}\,{\rm{Anode}})$$

where O_O_ represents an oxygen ion on a regular site, O^−2^ is oxygen anions and V_O_^++^ denotes oxygen vacancies with a double positive charge. If the anode is gold, platinum or another noble metal, it can receive the electrons from the oxygen anions and it may lead to the evolution of oxygen gas and leaves the oxygen vacancies behind^59^;

2 $${\bf{2}}{{\bf{O}}}^{-{\bf{2}}}\to {\bf{2}}{{\bf{e}}}^{-}+\frac{{\bf{1}}}{{\bf{2}}}{\bf{2}}{{\bf{O}}}_{{\bf{2}}}\uparrow $$

This reaction drives the formation of oxygen vacancies, but also generates oxygen in gaseous form. In order to see the impact of this oxygen evolution on our device, we fabricated similar resistive switching device with gold (Au) as top electrode. The fabricated Au/CoFe_2_O_4_/FTO did not show any resistive switching characteristics. On applying a positive bias on the top Au electrode a sudden change in current was observed, which was limited by similar compliance current as in Al/CoFe_2_O_4_/FTO device. This step is equivalent to electroforming process in Al/CoFe_2_O_4_/FTO device which creates a large number of oxygen vacancies in the CoFe_2_O_4_ layer. We analysed the top Au contact pad by optical microscope and scanning electron microscope before and after applying the positive bias (see supplementary information). The Au electrode surface before applying the bias was fairly smooth, but after applying the bias some bulges or bubbles/cracks were observed. The bubbles and cracks may come from the oxygen evolution on the application of bias. This oxygen can build up near the Au/CoFe_2_O_4_ interface and cause the damage of the resistive switching device by rupturing the electrode, which has been suggested earlier also^60^. If FTO electrode may be playing the dominating role in resistive switching and push/pull of oxygen ions occurs at bottom CFO/FTO interface, we would have observed the resistive switching behaviour in this Au/CFO/FTO device. Non-occurrence of resistive switching behaviour in Au/CFO/FTO confirms that in our fabricated device the top interface plays a dominating role. Shen at al. have observed similar bubbles/cracks for Pt/BaSrTiO_3_/SrRuO_3_ resistive switching device but not for W/BaSrTiO_3_/SrRuO_3_ device and indicated the role of top active electrode as a reservoir of oxygen ions^56^. The improved resistive switching of our samples with Al top electrodes is similar to the phenomenon observed in ZrO_2_ films with Ti top electrode^61^ and La_0.7_Ca_0.3_MnO_3_ films with samarium (Sm) top electrode^62^ which were also attributed to the existence of metal oxide layers formed near the top interface that acts like a source/sink of oxygen ions. As in our case, the top electrode is Al and on applying the positive bias to this, the incoming oxygen anions may oxidize it partially and form Al_2_O_3_ or more complex oxide of aluminum as per the following equation^63^;

3 $$\frac{{\bf{2}}}{{\bf{3}}}{\bf{Al}}+{\bf{O}}\to {{\bf{V}}}_{{\bf{o}}}^{++}+{\bf{2}}{{\bf{e}}}^{-}+\frac{{\bf{1}}}{{\bf{3}}}{\bf{A}}{{\bf{l}}}_{{\bf{2}}}{{\bf{O}}}_{{\bf{3}}}$$

Besides this, an oxygen diffusion layer may be formed at the interface due to the inter diffusion of oxygen from the oxide layer to the top metal electrode^64^. When the reverse voltage is applied the oxygen ions are pushed back to the CFO thin film. As the magnetic properties of CFO is dependent upon the oxygen vacancies^65^, the push/pull of oxygen from the CFO thin film is expected to change the magnetic properties of the device. In contrary to above observations some authors have suggested that for Al top and FTO/ITO bottom electrodes the role of the bottom electrode cannot be ignored easily and some oxygen vacancies/ions may be pushed/pulled from the bottom FTO/ITO electrode also^66–68^. In view of the above discussion, in our proposed model we suggest that Al and FTO both play roles in resistive switching behaviour, but the role of Al/CFO interface is dominating in the resistive switching process in our fabricated device.

The electroforming step is shown in Fig. 6(b) which takes the device from highly resistive pristine state to low resistance state (LRS) by providing a conducting channel of oxygen vacancies between the two electrodes. The electroforming process is not a sudden process, but a continuous increasing of electric field works on the oxygen anions and at a threshold voltage they leave their sites and start moving toward the anode due to a negative charge on them and oxygen vacancies are created on their sites. When these sufficient oxygen vacancies are created, these vacancies align and form a conducting channel. In our fabricated device, as a result of the inter diffusion of oxygen from CFO to top Al during the deposition of the Al layer and on applying the positive bias on the top electrode during the electroforming process, the top electrode may be oxidised partially and the interfacial oxygen diffusion layer can be formed as shown in Fig. 6(b). Figure 6(c) shows the Low Resistance State in which there is a stable conducting channel of oxygen vacancies with a weak link between the two electrodes. These oxygen vacancies can alter the magnetic properties of the CFO thin film as discussed above. On applying the reverse voltage on the electrodes, the oxygen ions are inserted back to the CFO layer, which neutralizes the oxygen vacancies partially, and this will result in the reset process (Fig. 6(d)). In High Resistance State the conducting channel of oxygen vacancies is broken as the maximum oxygen vacancies near the top electrode is refilled by the oxygen anions and convert them to regular oxygen sites. However a part of conducting oxygen vacancies' channel is still there so this state (high resistance state) is less resistive compared to pristine state but more resistive than LRS. Figure 6(f) shows the set process that takes the device from HRS to LRS. The partial refilling of the regular oxygen sites after the electroforming is also in favor of higher magnetization observed in the pristine state than high resistance state, which can be attributed to deficiency of oxygen vacancies in pristine state compared to that of the high resistance state. The low resistance state has more oxygen vacancies compared to both (pristine and high resistance) states that causes even lesser magnetization of this state. The proposed model successfully explains the modulation of magnetization for LRS and HRS in successive resistive switching cycles in the present device.

## Conclusion

In summary, we observed bipolar multifunctional Resistive Switching features in capacitor like Al/CoFe_2_O_4_/FTO device structure. Long retention capabilities, and good endurance performance have been demonstrated. The device shows different conduction mechanisms in different resistance states and in different voltage regimes and these conduction mechanisms have been explained in detail on the basis of resistance vs. temperature measurements and current vs. voltage curves. Different magnetic properties of the fabricated device have been observed in different resistance states, i.e. PS, LRS and HRS. A valence change model with an oxygen diffusion layer near the top Al electrode have been proposed to explain the observed magnetic and electric results. The existence of VCM in our Al/CFO/FTO resistive switching device has been confirmed by XPS analysis near the top Al/CFO interface. When a positive voltage bias is applied on the top electrode, the negatively charged oxygen ions from CFO migrate toward the low-electronegativity Al electrode causing the formation of an oxygen deficient conducting path in the CFO layer, which thus results in a decrease in the resistance of the device and causes switching from High Resistance State to Low Resistance State (the so-called SET process). On reversing the polarity of the applied voltage, the oxygen ions from the top electrode migrate toward the CFO layer. The drift in the oxygen ions toward the CFO make the device switch back to HRS (RESET process). The device is programmed and deprogrammed into LRS and HRS, through the forming and breaking of conducting channel of oxygen vacancies. The oxygen vacancies influences the valence cloud of the cations, that further affects the magnetic properties of the device, which becomes a strong evidence of electromigration of oxygen ions/vacancies during the resistive switching process and change the valence of lattice cations that confirms the presence valence change mechanism in resistive switching device. The coupling of resistive switching behaviour and magnetic modulation of the present device may have great potential in achieving future multifunctional memory devices.

## Methods

### Device fabrication

Our fabricated ReRAM device Al/CFO/FTO is consisted of CoFe_2_O_4_ thin film as an active layer, Aluminum (Al) as the top electrode and fluorine doped tin oxide (FTO) as the bottom electrode. The CoFe_2_O_4_ thin films (thickness ~200 nm) were prepared using sol–gel spin-coating method on FTO substrate. The Co(NO_3_)_2_.6H_2_O and Fe(NO_3_)_3_·9H_2_O were used as starting precursors which were separately dissolved in 2-methoxyethanol, and afterward mixed together with an appropriate molar ratio. The mixed solution with a total metal ion concentration of 0.2 M was then spin-coated on an FTO/glass substrate with a rotational speed of 3,000 rpm for 30 sec and repeated 4 times. After each coating, the film was dried at 150 °C for 10 min and finally annealed at 400 °C in air for 60 minutes. An ~100 nm thick Al film was evaporated onto the CFO thin film by electron beam physical vapor deposition system using a shadow mask, at a rate of 0.2 Å/s in the vacuum of 5 × 10^−6^ Torr. Similar steps were taken to fabricate Au/CFO/FTO device by using gold (Au) as a top electrode material instead of Aluminum (Al).

### Characterizations and measurements

Structural properties and phase purity of CFO active layer were investigated using X-ray diffraction and Raman spectra (see supplementary information). The I–V characteristics of the device were recorded at room temperature using a Keithley 2400 source meter with LabVIEW software. The switching measurements were done in voltage sweep mode, and the bias was defined as positive when the electrical current flowed from top (Al) electrode to bottom (FTO) electrode. A compliance current of 100 mA was used to prevent the device from a permanent breakdown. Resistance vs temperature (R-T) measurements were performed in temperature range from 300 to 475 K. The magnetic measurements of the fabricated device were performed using the Alternating Gradient Magnetometer (Princeton Micro Mag 2900) in the magnetic field range of -1 to 1 Tesla. X-ray photoelectron spectroscopy (XPS) measurements were carried out using an EA 125 electron spectrometer manufactured by OMICRON Nanotechnology GmbH (Germany), with Al-Kα radiation (1486.7 eV).

## Electronic supplementary material

Supplemaentary info
