# Supplementary material for: Valence Change Bipolar Resistive Switching Accompanied With Magnetization Switching in CoFe2O4 Thin Film
Source: Sci Rep. 2017 Sep 29;7:12427. doi: 10.1038/s41598-017-12579-x (PMC5622061; doi:10.1038/s41598-017-12579-x)
Supplement: Supplementary file 1 — Supplemaentary info [file 41598_2017_12579_MOESM1_ESM.pdf]

Valence Change Bipolar Resistive Switching Accompanied With Magnetization Switching in $CoFe_2O_4$ Thin Film.

Sandeep Munjal and Neeraj Khare*

Department of Physics, Indian Institute of Technology Delhi, Hauz Khas, New Delhi-110016, India.

Supporting information

Structural properties and phase purity of $CoFe_2O_4$ thin film were investigated using X-ray diffraction and Raman spectra

XRD pattern of $CoFe_2O_4$ thin film is shown in **Fig. S1**. The peaks observed at 2θ = 30.26°, 35.53°, 37.15°, 43.21°, 53.62°, 57.18°, 62.73° and 74.72° corresponded to (220), (311), (222) (400), (422), (511), (440) and (530) planes of spinel $CoFe_2O_4$ respectively (JCPDS No. 22-1086), which confirmed the formation of single phase cubic spinel structure of $CoFe_2O_4$ thin film.[1]

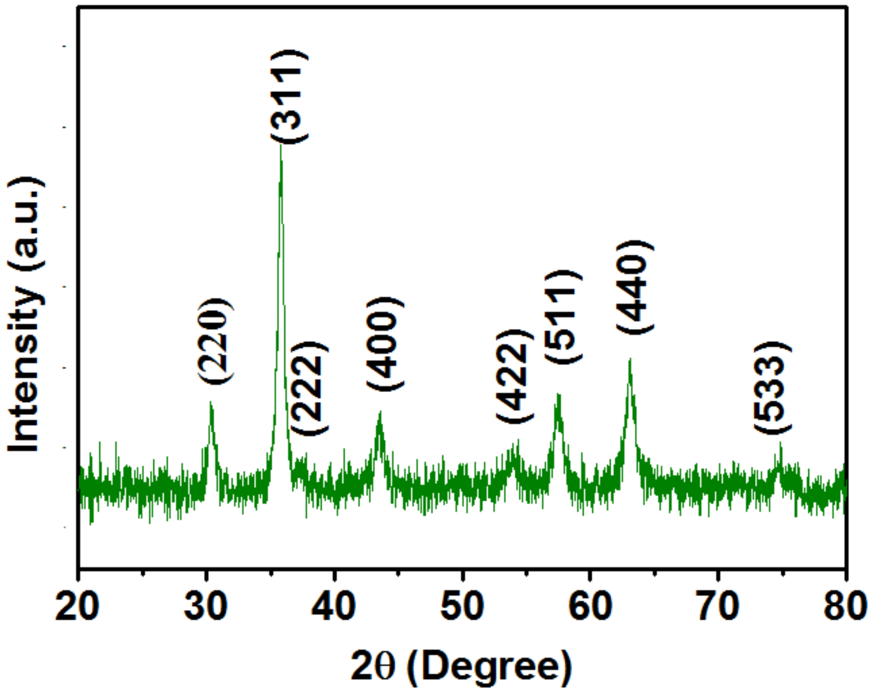

Fig. S1 XRD pattern of $CoFe_2O_4$ thin film.

The single phase growth of $CoFe_2O_4$ thin film was also confirmed by Raman spectra at room temperature (Horiba LabRAM HR Evolution). Raman spectra of $CoFe_2O_4$ thin was recorded using argon laser source (excited at 514 nm and 10 mW), and shown in **Fig. S2**. The Raman peaks observed near 305, 352, 467, 551, 615, and 671 cm^{-1} corresponded to optical active Raman modes (A_{1g} + E_g + $3T_{2g}$) with space group $Fd\bar{3}m$ of cubic spinel $CoFe_2O_4$ structure.[2]

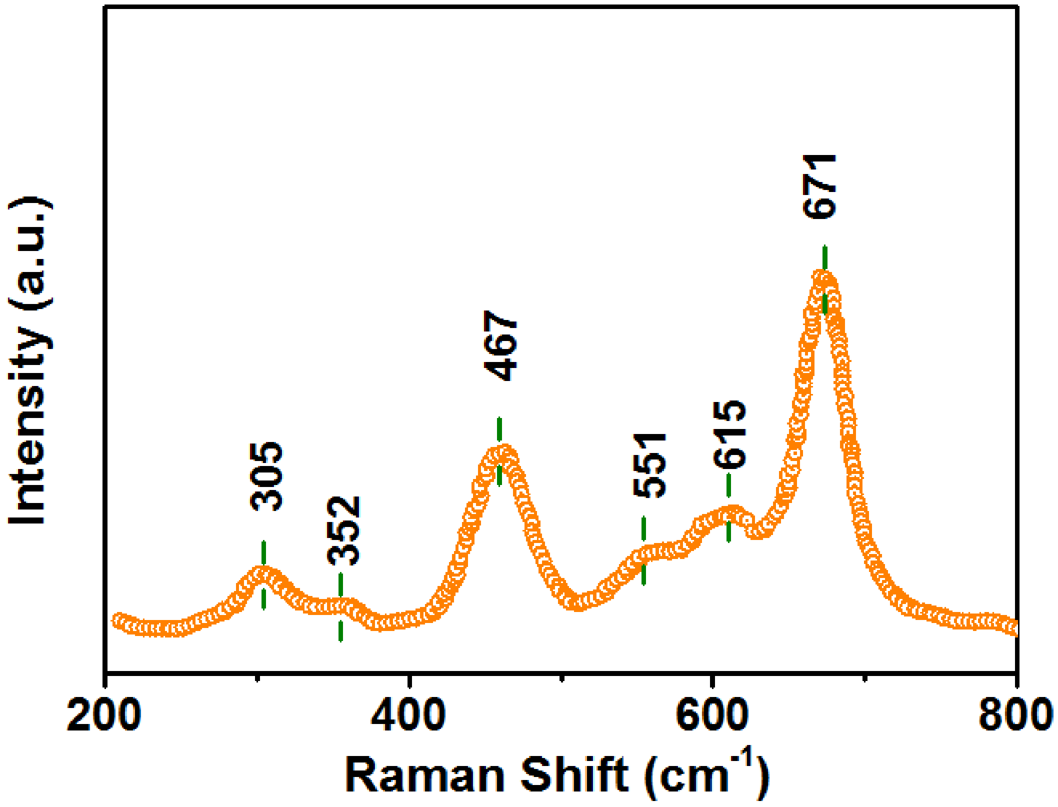

Fig. S2 Raman spectra of $CoFe_2O_4$ thin film.

To get more insight into the Resistive Switching mechanism of our device, we conducted X-ray photoelectron spectroscopy (XPS) studies of Al/CFO/FTO structure in Pristine, Low Resistance State and High Resistance State.

For pristine sample a small peak correspond to Al–O was observed in the XPS spectra of the Al 2p signal near the interface, which indicates that some oxygen atoms of CFO layer near the interface are taken away by Al atoms. For the device in Low Resistance State after electroforming XPS spectra of the sample reveals that near the Al-CFO interface the peak area of Al–O component was significantly higher than Al-O component observed in Pristine state indicating that during the switching cycles when device was switched ON, on applying positive bias, at the anode, the oxygen ions moves under the effect of applied electric field and convert Al of top electrode partially into Al_2O_3 or other complex oxides. However, for High Resistance state device, Al-O component near interface was comparable to that of Low Resistance State suggesting the stability of aluminium oxide formed during the initial voltage sweep.

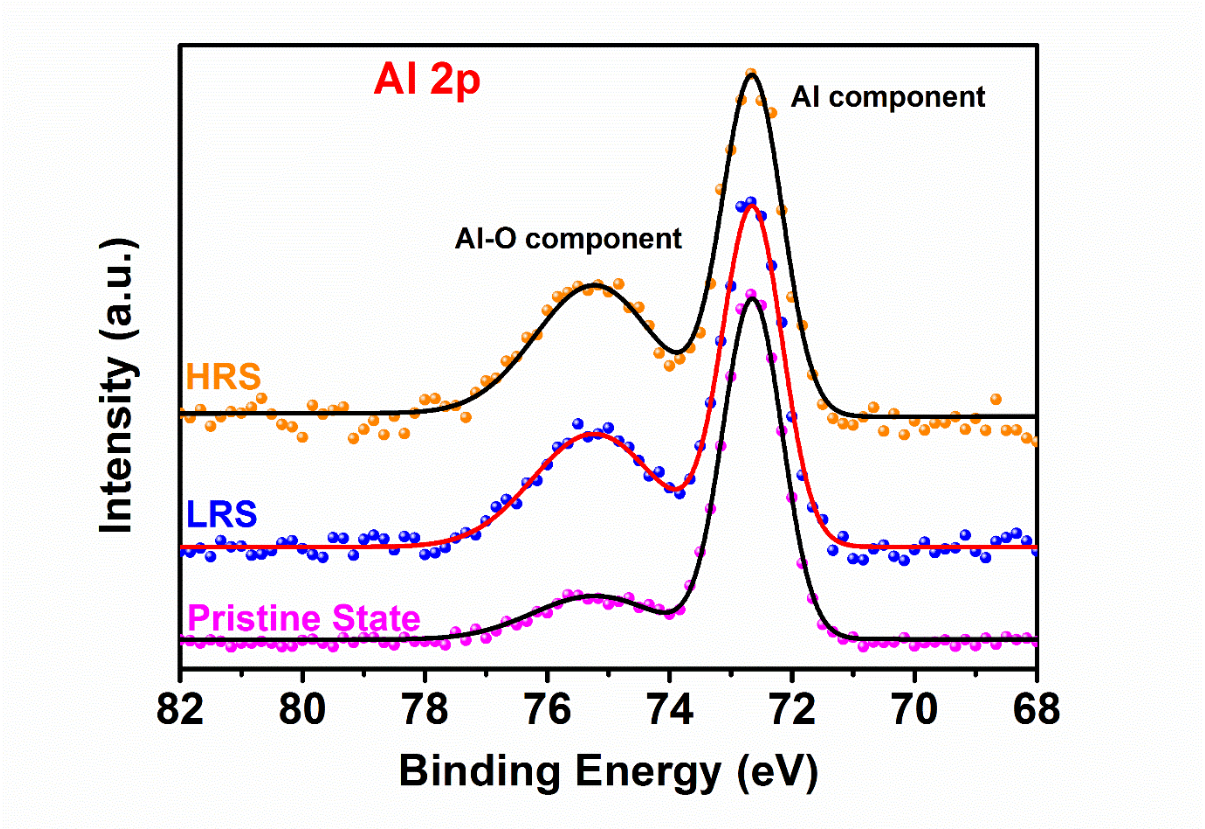

Fig. S3. XPS spectra of Al 2p near the Al/CFO interface of the device.

No significant change was observed in the Fe 2p XPS spectrum of the device in pristine, Low Resistance and High resistance state (**Fig. S4.).**

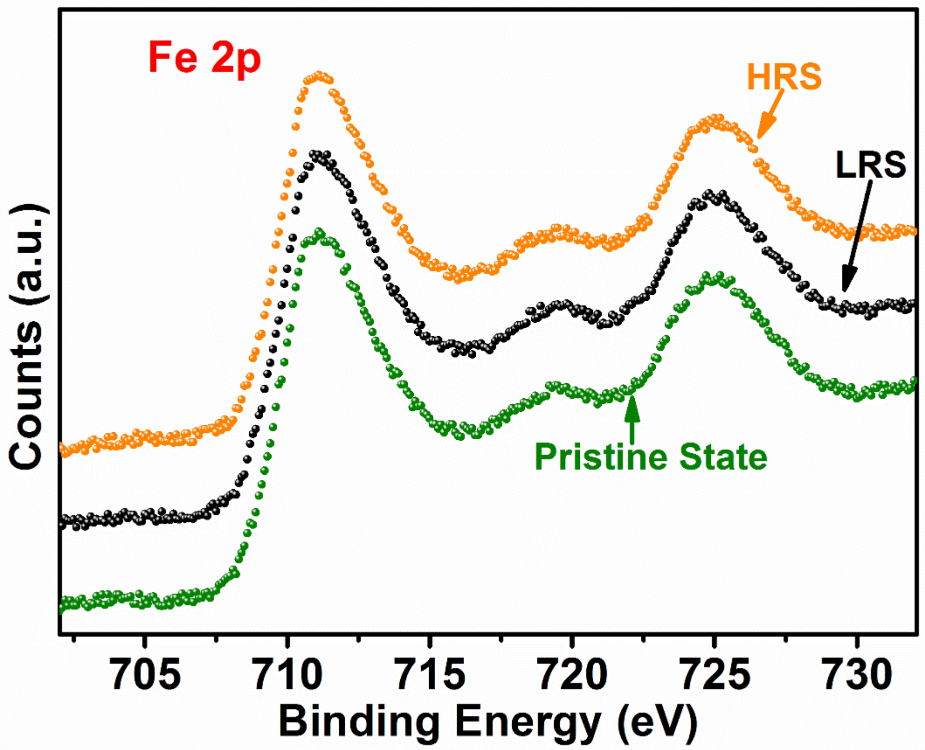

Fig. S4. XPS spectra of Fe 2 p at the Al/CFO interface of the device.

We have performed experiments to see the dependence of resistance of the device in LRS and HRS on the area of the top Al contact pad. We observed that the resistance of the device in LRS and HRS is independent of the contact pad area for the variation of pad area from ~ 0.05 mm^2 to 2 mm^2 (Fig. S5). This confirms that the resistive switching in our fabricated Al/$CoFe_2O_4$/FTO is not of interface type.

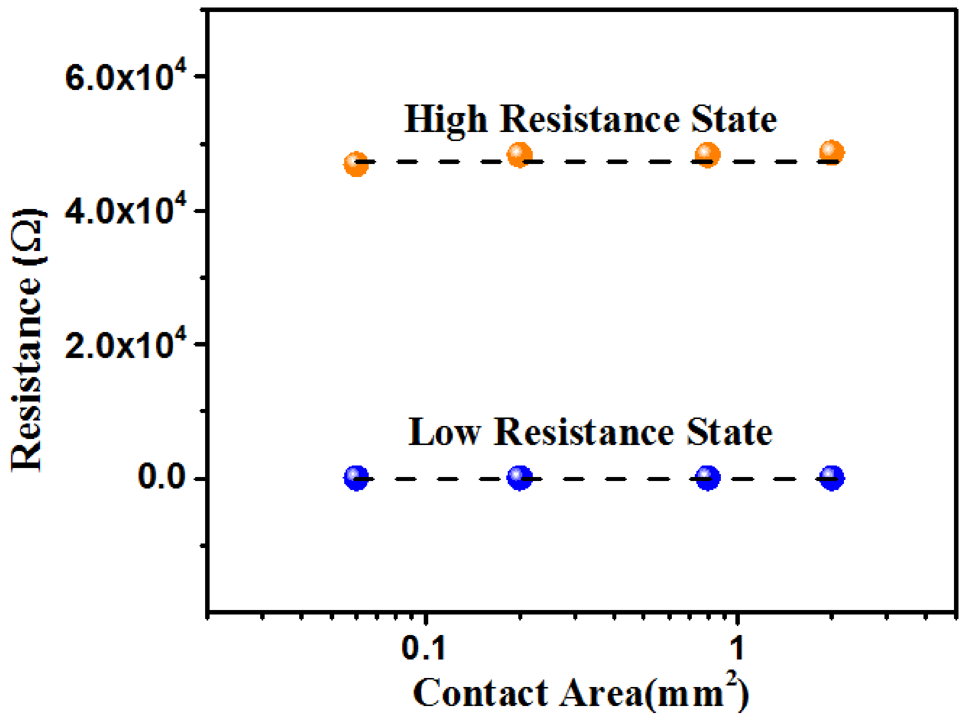

Fig. S5. Top contact area dependence of resistance in LRS and HRS.

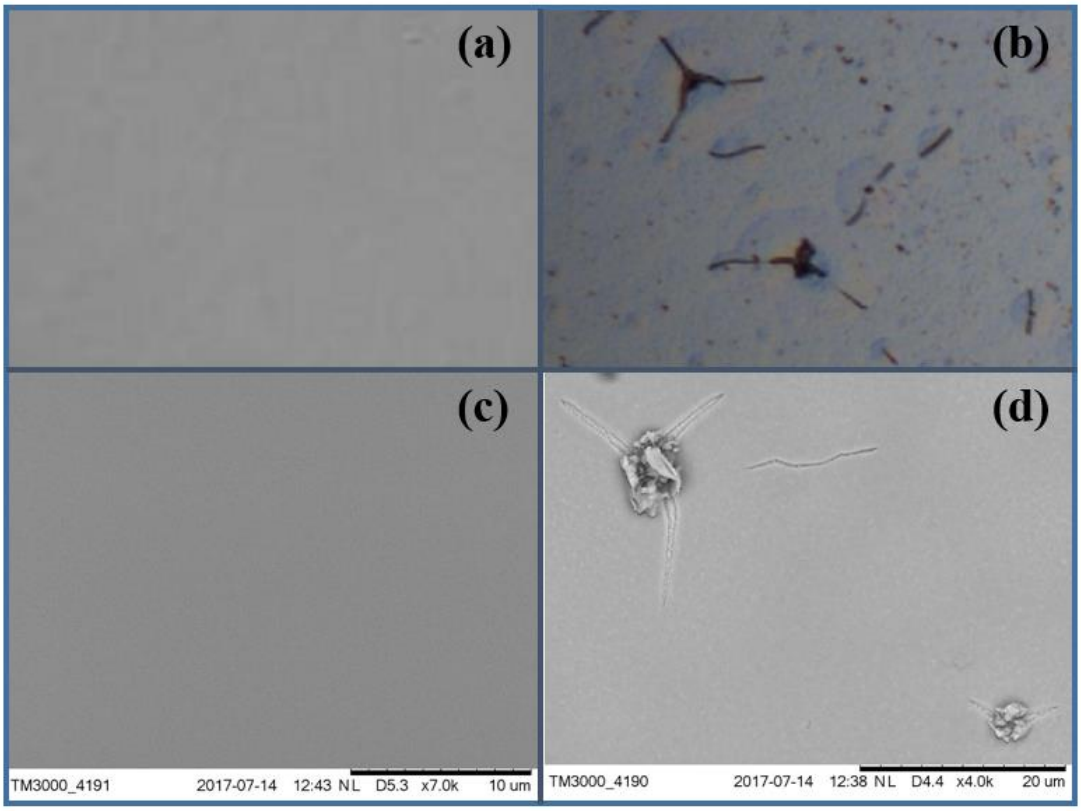

Fig. S6. ((a)-(b)) Optical microscopy and ((c)-(d)) scanning electron microscopy images of the top Au electrode of Au/CFO/FTO device ((a) and (c)) before and ((b) and (d)) after applying positive bias.

References

1. He, Y. *et al.*, *ACS Appl. Mater. Interfaces* **8,** 7683–7690 (2016).
2. Singh, S., Munjal, S. & Khare, N., *J. Magn. Magn. Mater.* **386,** 69–73 (2015).
